# Pretherapeutic Assessment of Pancreatic Cancer: Comparison of FDG PET/CT Plus Delayed PET/MR and Contrast-Enhanced CT/MR

**DOI:** 10.3389/fonc.2021.790462

**Published:** 2022-01-14

**Authors:** Zaizhu Zhang, Nina Zhou, Xiaoyi Guo, Nan Li, Hua Zhu, Zhi Yang

**Affiliations:** Key Laboratory of Carcinogenesis and Translational Research (Ministry of Education/Beijing), NMPA Key Laboratory for Research and Evaluation of Radiopharmaceuticals (National Medical Products Administration), Department of Nuclear Medicine; Peking University Cancer Hospital & Institute, Beijing, China

**Keywords:** pancreatic cancer, contrast-enhanced CT/MR, FDG, PET/CT, PET/MR

## Abstract

**Purpose:**

This study aims to determine the diagnostic performance of whole-body FDG PET/CT plus delayed abdomen PET/MR imaging in the pretherapeutic assessment of pancreatic cancer in comparison with that of contrast-enhanced (CE)-CT/MR imaging.

**Materials and Methods:**

Forty patients with pancreatic cancer underwent nonenhanced whole-body FDG PET/CT, delayed abdomen PET/MR imaging, and CE-CT/MR imaging. Two nuclear medicine physicians independently reviewed these images and discussed to reach a consensus, determining tumor resectability according to a 5-point scale, N stage (N0 or N positive), and M stage (M0 or M1). With use of clinical-surgical-pathologic findings as the reference standard, diagnostic performances of the two imaging sets were compared by using the McNemar test.

**Results:**

The diagnostic performance of FDG PET/CT plus delayed PET/MR imaging was not significantly different from that of CE-CT/MR imaging in the assessment of tumor resectability [area under the receiver operating characteristic curve: 0.927 vs. 0.925 (*p* = 0.975)], N stage (accuracy: 80% (16 of 20 patients) vs. 55% (11 of 20 patients), *p* = 0.125), and M stage (accuracy: 100% (40 of 40 patients) vs. 93% (37 of 40 patients), *p* = 0.250). Moreover, 14 of 40 patients had liver metastases. The number of liver metastases detected by CE-CT/MR imaging, PET/CT, and PET/MR imaging were 33, 18, and 61, respectively. Compared with CE-CT/MR imaging, PET/MR imaging resulted in additional findings of more liver metastases in 9/14 patients, of which 3 patients were upstaged. Compared with PET/CT, PET/MR imaging resulted in additional findings of more liver metastases in 12/14 patients, of which 6 patients were upstaged.

**Conclusions:**

Although FDG PET/CT plus delayed PET/MR imaging showed a diagnostic performance similar to that of CE-CT/MR imaging in the pretherapeutic assessment of the resectability and staging of pancreatic tumors, it still has potential as the more efficient and reasonable work-up approach for the additional value of metastatic information provided by delayed PET/MR imaging.

## Introduction

Pancreatic cancer remains a highly lethal malignancy, with a 5-year survival rate of less than 10%, and is the seventh most common cause of cancer death in both men and women worldwide (1–3). The only potential curative treatment for pancreatic cancer is radical surgical resection (4). However, at the time of initial staging work-up, approximately 80%–85% of patients present with either unresectable or metastatic disease owing to lack of early and specific symptoms when the cancer is still localized, and high metastasis rate (1, 3, 4). Given this, imaging examinations are destined to play an irreplaceable role in early diagnosis and accurate staging, which are crucial for choosing appropriate therapy strategy and preventing unnecessary surgery (5, 6).

Various anatomical imaging modalities including contrast-enhanced computed tomography (CE-CT), magnetic resonance (MR) imaging, and endoscopic ultrasonography are routinely used in the initial staging work-up of pancreatic cancer (5, 7), with CE-CT considered the most commonly used and best validated imaging modality (3, 7). In addition to anatomical imaging examinations, another modality that has shown potential is fluorine 18 fluorodeoxyglucose (FDG) positron emission tomography (PET)/CT, which is sensitive for initial TNM staging (8), evaluation of treatment response (9), detection of recurrence (10), and prediction of treatment efficacy and clinical outcome and has been reported to improve the detection of occult metastases, ultimately sparing these patients from unnecessary surgery (11–13). Recently, PET/MR, as an emerging imaging technology, provides both multiparametric functional imaging, including diffusion-weighted imaging (DWI), and metabolic information from PET, with many potential advantages over PET/CT, including inherently lower radiation exposure, higher soft-tissue contrast, and multiparametric imaging capabilities (5, 7, 14–19).

Coincidentally, due to the different advantages of each imaging modality, multiple imaging modalities are being increasingly used in patients with pancreatic cancer, and this multistep examination process probably leads to delayed surgical treatment for resectable diseases (20, 21). Hence, developing the more efficient and reasonable work-up approach is of great clinical significance for patients with pancreatic cancer. Indeed, a previous study has demonstrated a similar diagnostic performance between FDG PET/MR and PET/CT plus CE-CT in the preoperative evaluation of the resectability and staging of pancreatic tumors (7). To our knowledge, however, the comparison of diagnostic performance between nonenhanced whole-body FDG PET/CT plus delayed abdomen PET/MR and CE-CT/MR for tumor staging and resectability of pancreatic tumors has not been reported. Thus, the purpose of this study was to compare the diagnostic performance of nonenhanced whole-body FDG PET/CT plus delayed abdomen PET/MR in evaluating tumor staging and resectability of pancreatic cancer with that of the conventional CE-CT/MR, which would be useful for simplifying the multistep process and even choosing the more efficient and reasonable work-up flow.

## Materials and Methods

### Patients

This study was performed under a single-center prospective imaging protocol and was approved by the Medical Ethics Committee of Peking University Cancer Hospital (ethical approval No. 2018KT110-GZ01). All patients provided signed informed consent before the examinations.

From December 2019 to April 2021, 67 consecutive patients (33 men and 34 women; mean age ± standard deviation, 60.5 years ± 10.9) with histologically confirmed or suspected pancreatic cancer were prospectively and consecutively enrolled in this study. These candidates took a whole-body nonenhanced FDG PET/CT scan first, followed by a delayed abdomen PET/MR scan with a 120–180-min interval. The key eligibility criteria were as follows: (a) confirmatory evidence with either histology or metastases at follow-up imaging; (b) patients have undergone chest CT, abdomen, and pelvis CE-CT/MR, and the interval time between PET and CT/MR was less than 30 days; and (c) no contraindication to PET/MR imaging. Additionally, patients with any of the following conditions were excluded: (a) age <18 or >80 years old and (b) insufficient follow-up to confirm the reference standard.

### Image Acquisition

#### ^18^F-FDG PET/CT

Imaging was performed using a PET/CT scanner (Biograph64, SIEMENS, Erlangen, Germany) operated in 3D Flow Motion (bed entry speed 1 mm/s) from the apex of the skull to the mid-thigh, with a PET axial field of view of 21.6 cm. The PET images were reconstructed by the TrueX + TOF method offered by the vendor. Low-dose CT scans were acquired in CARE Dose4D mode (120 kV, 3.0 mm slice thickness). The patients were instructed to fast for at least 6 h before ^18^F-FDG injection. In all cases, the serum glucose concentration met our institutional requirement (≤140 mg/dl). The injected activity was 3.7 MBq/kg, and the time from injection to scan was 60 min.

#### ^18^F-FDG PET/MR Imaging

^18^F-FDG PET/MR imaging was performed on an integrated 3.0 T time-of-flight PET/MR scanner (uPMR790, UIH, Shanghai, China). The scan started at 120 min (range: 120–180 min) after FDG-administration. Each patient underwent the same protocol as described in the following. Body array coil was placed around the individual and covered the entire liver and pancreas. Respiratory gating was used in MR acquisition whenever possible. PET reconstruction was conducted using a 3D-OSEM (Ordered Subsets Expectation-Maximization) algorithm applied on a 256 × 256 matrix. A four-compartment-model attenuation map (μ-map) automatically generated based on a water-fat-imaging MR sequence was used for PET attenuation correction. The PET images were smoothed by a Gaussian filter with 3 mm full width at half maximum (FWHM). The MR sequences were performed simultaneously with PET acquisition, including T1-weighted imaging (T1WI), T2-weighted imaging (T2WI), fat-suppressed T2WI, and DWI. The mean scan time for PET/MR was 20 ± 6 min.

### Image Interpretation

**Figure 1:**
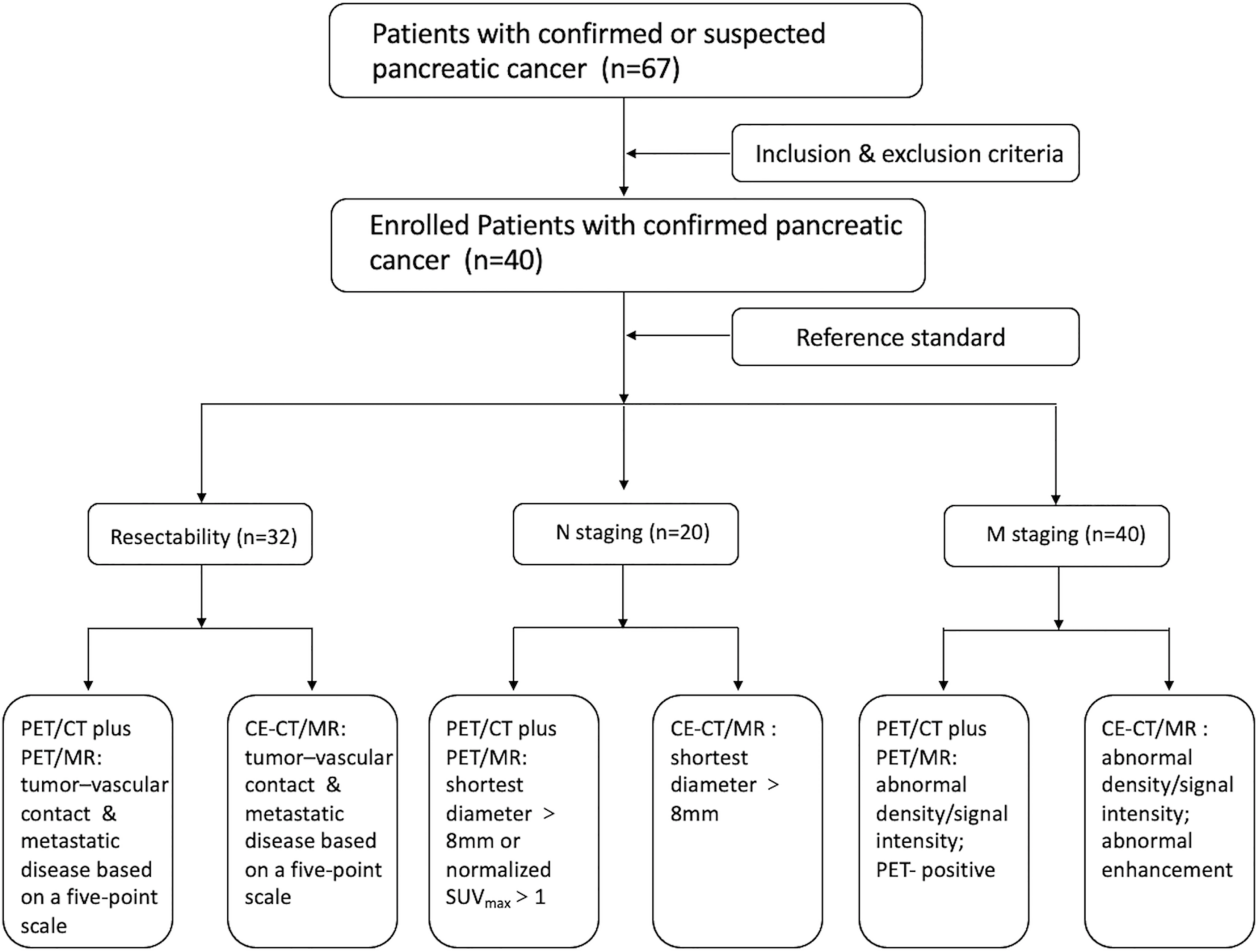
The flow chart of the study.

All images were reviewed using our local picture archiving and communication system (PACS). To avoid bias, two experienced nuclear medicine physicians independently analyzed the nonenhanced whole-body FDG PET/CT, delayed abdomen PET/MR images, and CE-CT/MR images, and the results were discussed to reach a consensus (**Figure 1**).

#### Assessment of Tumor Resectability

The reviewers determined the resectability of pancreatic tumors on the basis of tumor location, tumor–vascular contact, adjacent organ invasion, and metastatic disease based on a 5-point scale, as follows: 5, definitely resectable; 4, probably resectable; 3, equivocal; 2, probably unresectable; and 1, definitely unresectable (7). Unresectable disease was further specified as locally advanced disease (i.e., pancreatic cancer without distant metastasis but with unresectable vascular invasion) or pancreatic cancer with distant metastasis (22, 23).

#### Determination of N Stage

The maximum standardized uptake value (SUVmax) of the lymph nodes was calculated in the same lesion on both FDG PET/CT and delayed PET/MR images. Regions of interest were drawn around foci with increased uptake in the transaxial slices, and an original SUVmax was automatically obtained. To ensure SUVmax relatively comparable, the original SUVmax was normalized by the following formula (24):


$$\text{Normalized SUV}\max=\text{Original SUV}\max{\text{/SUV}}_{\text{bkgd}}$$

SUV_bkgd_ refers to average SUV of the descending aorta.

Positive lymph nodes were determined on the basis of their size and quantitative assessment of PET images. If the largest regional lymph node was at least 8 mm in its shortest diameter (7) or the uptake greater than the blood pool (normalized SUVmax >1) at quantitative assessment of early or delayed PET scans (25), the patient would be considered node positive, but otherwise as negative.

#### Determination of M Stage

At PET/MR imaging, the lesions were rated as metastases when at least two of the three following criteria were met: (a) abnormal signal intensity on T2WI, (b) diffusion restriction on DWI with *b* values of 800 s/mm^2^, and (c) positivity on PET scans at visual assessment. At PET/CT imaging, the lesions were defined as metastases when PET had positive uptake foci with abnormal density on CT. At CE-CT/MR imaging, the lesions were determined as metastases when they had abnormal density/signal intensity with abnormal enhancement.

#### Reference Standard

The reference standard for tumor resectability was based on surgical records, pathological findings, and imaging-based decisions. In patients who underwent surgery, tumor resectability was assessed in light of surgical records and pathologic reports, as follows: R0 resection (complete tumor resection with a negative resection margin) was defined as resectable and R1 resection (uncomplete tumor resection with a microscopically positive resection margin) and R2 resection (uncomplete tumor resection with a macroscopically positive resection margin) as no resection of the pancreatic mass due to unresectability confirmed during surgery, and presence of pathologically confirmed distant metastasis were defined as unresectable (7). Additionally, if a patient had distant metastases and/or locally unresectable tumor at preoperative imaging and did not undergo surgery based on a multidisciplinary conference, the patient was also considered unresectable (7). For N staging, the reference standard was determined by the pathologic findings in patients who underwent regional lymph node dissection (7). For M staging, the reference standard of M0 was determined with histopathologic findings or follow-up images, whereas that of M1 was determined with histopathologic results or imaging-based decisions made by means of a multidisciplinary conference (7).

### Statistical Analysis

Diagnostic performances for per-patient resectability, N staging, and M staging were evaluated in patients by using standards of reference. Tumor resectability was evaluated with empirical receiver operating characteristic curve analysis based on a 5-point confidence scale. The area under the receiver operating characteristic curve (AUC) was regarded as an indicator of diagnostic performance, and areas under the receiver operating characteristic curve values of PET/CT plus delayed PET/MR imaging and CE-CT/MR imaging were compared by using the *z*-test. Furthermore, the examinations given scores of 4 or 5 (probably or definitely resectable) were defined as resectable. For tumor resectability, N stage and M stage, sensitivity, specificity, and accuracy were compared between PET/CT plus delayed PET/MR imaging and CE-CT/MR imaging by using the McNemar test. Moreover, for per-lesion analysis, the numbers of liver metastases detected by PET/CT plus PET/MR imaging were compared with only PET/CT or CE-CT/MR imaging. All statistical analyses were performed using MedCalc, version 20.0.4 (MedCalc Software, Mariakerke, Belgium). Two-tailed *p* < 0.05 was considered to indicate a significant difference.

## Results

### Patient Characteristics

On the basis of the inclusion and exclusion criteria, 40 patients (23 men and 17 women; mean age ± standard deviation, 58.9 years ± 9.1) were enrolled finally, of which 24 patients underwent CE-CT scan, and the remaining 16 patients underwent CE-MR scan. Among the 40 patients, the tumor resectability was confirmed in 32 patients (resectable, *n* = 17; unresectable, *n* = 15) based on surgery and distant metastases. Tumor resectability could not be confirmed in 8 patients, which were lost to follow-up. N stage was confirmed with histopathologic findings in 20 patients who underwent surgical resection for pancreatic cancer (node negative, *n* = 7; node positive, *n* = 13), and M stage was confirmed in 40 patients (M0, *n* = 23; M1, *n* = 17) by means of histopathologic reports (M0, *n* = 19; M1, *n* = 5) or imaging-based diagnosis (M0, *n* = 4; M1, *n* = 12). The M1 stage results include 14 patients with hepatic metastases confirmed with surgery (*n* = 1), biopsy (*n* = 3) and imaging-based diagnosis (*n* = 10), 5 patients with peritoneal seeding metastases found with biopsy (*n* = 1) and imaging-based diagnosis (*n* = 4), and three patients with an imaging-based diagnosis of pulmonary metastases. None of them underwent neoadjuvant chemo/chemoradiotherapy before these imaging examinations. The remaining basic characteristics, like tumor size, tumor location, and tumor SUVmax, are presented in **Table 1**.

**Table 1:** Basic information of the 40 patients with pancreatic cancer.

Characteristic	Value
Age (years)	58.9 ± 9.1 (40–75)
Gender (M/F)	23/17
Tumor number (*n*)	40
Maximum lesion diameter in axial section (cm)	3.5 + 2.2 (0.9–13.7)
Location	
Head	20 (50)
Neck	3 (7.5)
Body	10 (25)
Tail	7 (17.5)
Tumor SUVmax	
PET/CT	6.2 ± 2.6 (0.9–12.2)
PET/MR	4.9 ± 2.3 (0.9–9.3)
Tumor resectability (*n*)	32
Resectable	17 (53.1)
Unresectable	15 (46.9)
N stage (*n*)	20
Positive	13 (65)
Negative	7 (35)
M stage (*n*)	40
M0	23 (57.5)
M1	17 (42.5)

The data presented are means ± standard deviation (range) or number (percentage) of patients.

### Assessment of Tumor Resectability and N and M Staging

#### Tumor Resectability (*n* = 32)

**Figure 2:**
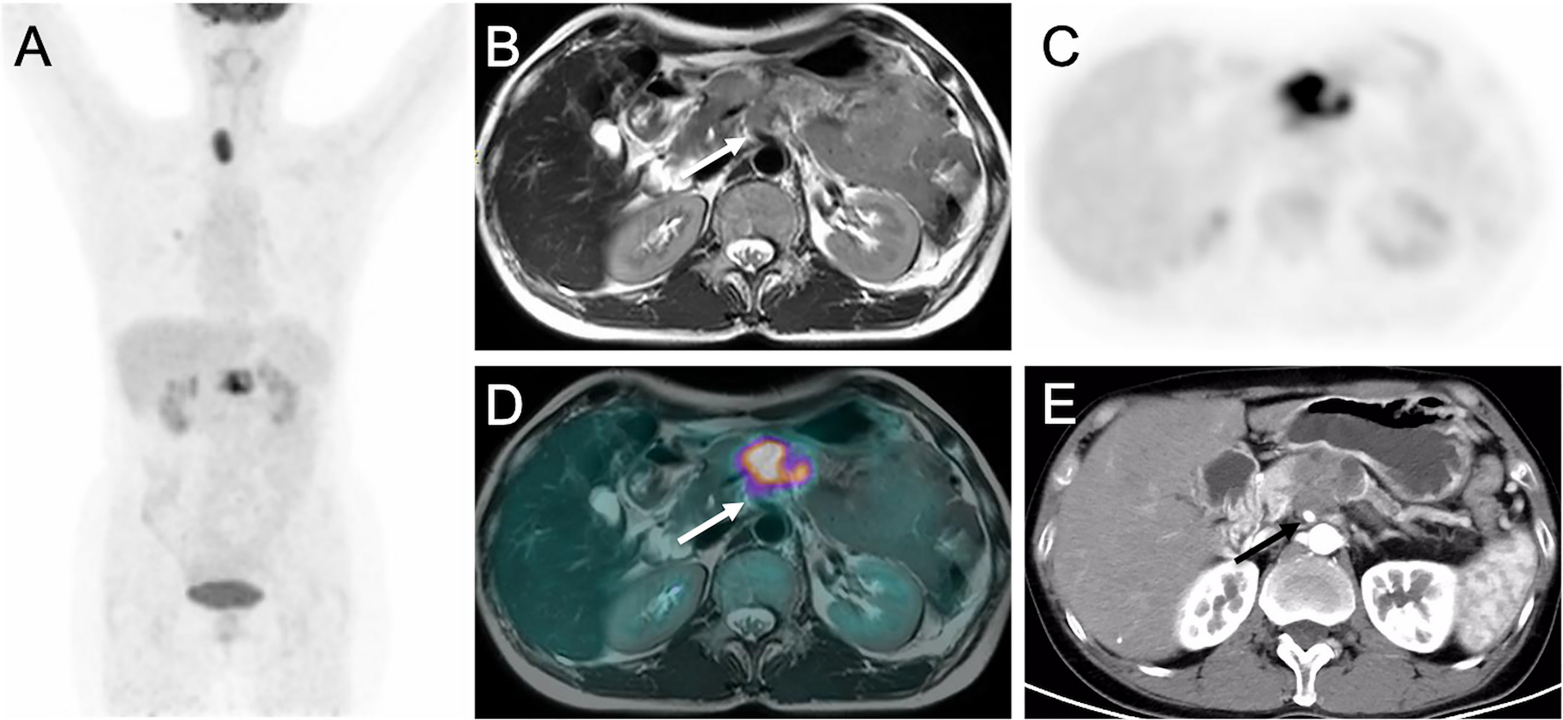
Images of pancreatic ductal adenocarcinoma in 52-year-old woman with vascular invasion. **(A)** MIP from PET/CT showing increased uptake in pancreas. **(B)** T2WI, **(C)** Delayed PET image, **(D)** Corresponding PET/MR imaging fusion image and **(E)** Arterial phase CT image show the mass in the body of pancreas encasing superior mesenteric artery (arrows).

For the evaluation of per-patient tumor resectability, there were no significant differences in the AUC between PET/CT plus delayed PET/MR imaging and CE-CT/MR imaging [0.927 vs. 0.925 (*p* = 0.975)] (**Table 2**). When scores of 4 and 5 (i.e., probably or definitely resectable) were categorized as indicating an imaging diagnosis of tumor resectability, PET/CT plus delayed PET/MR imaging and CE-CT/MR imaging showed the same accuracies of 88% (28 of 32 patients) versus 88% (28 of 32 patients), without a significant difference (*p* = 1.000) (**Table 2**; **Figure 2**). Moreover, PET/CT plus delayed PET/MR imaging showed the same sensitivity and specificity as CE-CT/MR imaging (82% (14 of 17 patients) vs. 82% (14 of 17 patients), and 93% (14 of 15 patients) vs. 93% (14 of 15 patients), respectively), although there were no statistically significant differences (**Table 2**).

**Table 2:** Diagnostic performance of PET/CT plus delayed PET/MR imaging and CE-CT/MR imaging in the assessment of tumor resectability.

Modality	$A_{z}^{a}$	Sensitivity (%)^b^	Specificity (%)^b^	Accuracy (%)^b^
PET/CT plus PET/MR	0.927 (0.778, 0.989)	82 (14/17)	93 (14/15)	88 (28/32)
CE-CT/MR	0.925 (0.775, 0.988)	82 (14/17)	93 (14/15)	88 (28/32)
*p*-value	0.975	NA	1.000	1.000

NA, not assessable; A_z_, area under the receiver operating characteristic curve.

a Data were calculated with the z-test. Numbers in parentheses are 95% CIs.

b Calculated with the McNemar’s test. Numbers in parentheses are numbers of patients.

#### N Staging (*n* = 20)

For N staging, diagnostic accuracies were not significantly different between the two image sets (80% (16 of 20 patients) with PET/CT plus delayed PET/MR imaging vs. 55% (11 of 20 patients) with CE-CT/MR imaging (*p* = 0.125) (**Table 3**). In the depiction of any regional lymph node metastasis per patient, PET/CT plus delayed PET/MR imaging showed higher sensitivity [92% (12 of 13 patients) vs. 46% (6 of 13 patients)], with a statistically significant difference (*p* = 0.031) and lower specificity [57% (4 of 7 patients) vs. 71% (5 of 7 patients)] than CE-CT/MR imaging, without a statistically significant difference (*p* = 1.000) (**Table 3**).

**Table 3:** Diagnostic performance of PET/CT plus delayed PET/MR imaging and CE-CT/MR imaging in the assessment of N and M stages.

Modality	N staging (%)			M staging (%)		
	Sensitivity	Specificity	Accuracy	Sensitivity	Specificity	Accuracy
PET/CT plus PET/MR	92 (12/13)	57 (4/7)	80 (16/20)	100 (17/17)	100 (23/23)	100 (40/40)
CE-CT/MR	46 (6/13)	71 (5/7)	55 (11/20)	82 (14/17)	100 (23/23)	93 (37/40)
*p*-value	0.031	1.000	0.125	0.250	NA	0.250

NA, not assessable.

p-values were calculated by using the McNemar’s test. Data in parentheses are numbers of patients used to calculate percentages.

#### M Staging (*n* = 40)

For M staging, PET/CT plus delayed PET/MR imaging and CE-CT/MR imaging demonstrated sensitivities of 100% (17 of 17 patients) and 82% (14 of 17 patients), without a statistically significant difference (*p* = 0.250) (**Table 3**). Both imaging sets showed high specificity [100% (23 of 23 patients)] for M staging. In addition, diagnostic accuracies were not significantly different between the two image sets (100% (40 of 40 patients) with PET/CT plus delayed PET/MR imaging vs. 93% (37 of 40 patients) with CE-CT/MR imaging (*p* = 0.250) (**Table 3**).

#### Additional Value of PET/MR in Patients With Liver Metastases (*n* = 14)

**Figure 3:**
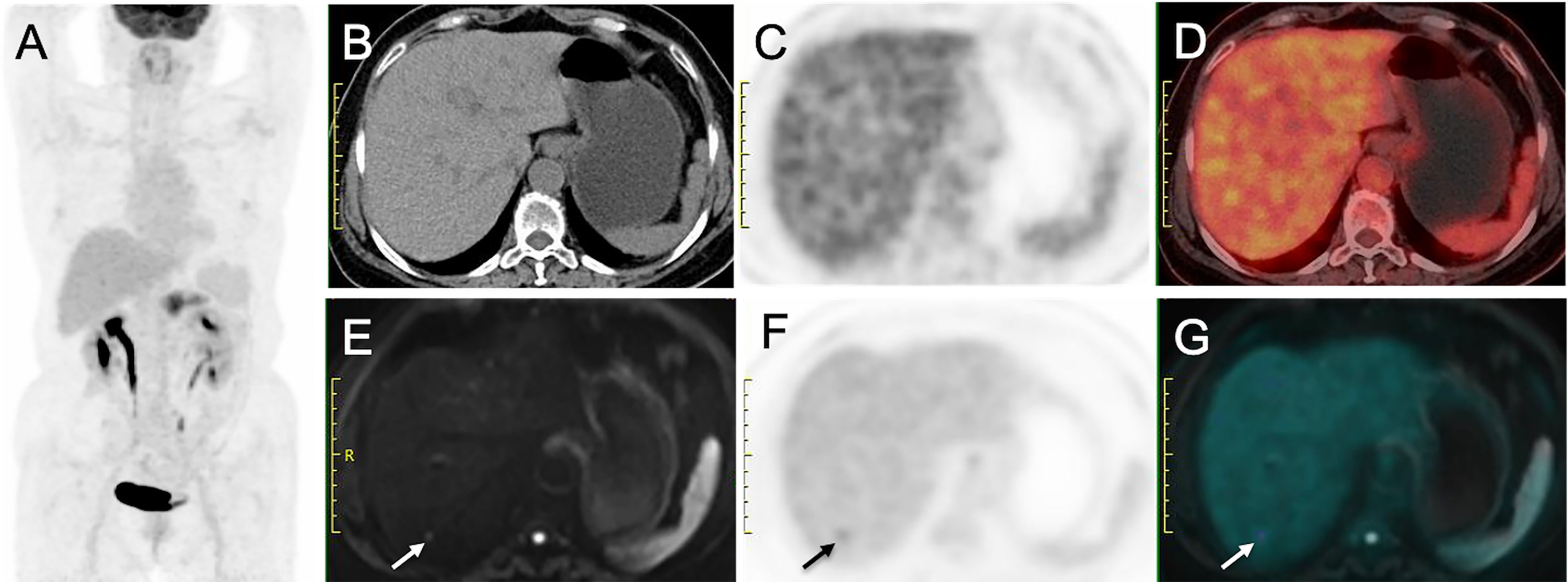
Liver metastasis detected on PET/MRI but not on PET/CT. **(A)** MIP from PET/CT showing increased uptake in pancreas. **(B)** Nonenhanced CT image, **(C)** Early PET image and **(D)** PET/CT fusion image show no hypoattenuating or hypermetabolic lesion in liver. **(E)** DW image (b = 800 sec/mm2) shows a nodule with restricted diffusion (arrow). **(F)** Delayed PET image shows a hypermetabolic lesion in liver. **(G)** Corresponding PET/MR imaging fusion image shows the nodule with both hyperintense and hypermetabolism. This patient was diagnosed as having stage M1 disease on PET/MR imaging but stage M0 disease on PET/CT.

**Figure 4:**
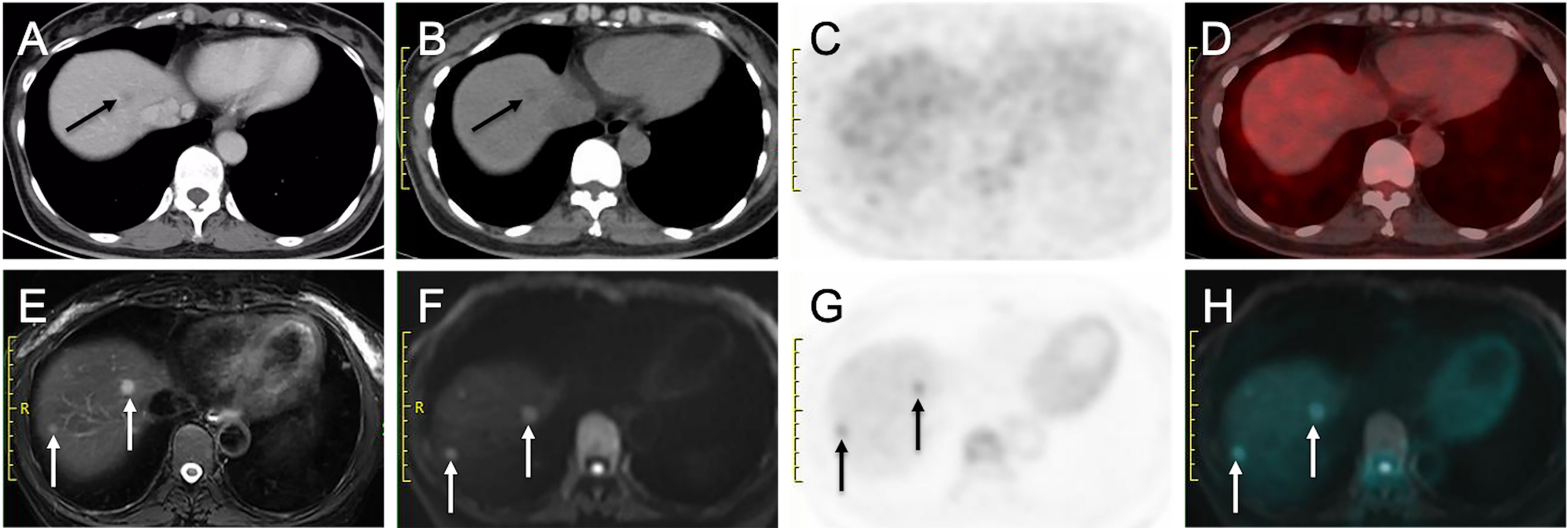
More liver metastases detected on delayed PET/MRI than on CE-CT and early PET/CT. **(A)** Venous phase CT image shows a hypo-enhanced nodule in liver (arrow). **(B)** Nonenhanced CT image shows a hypoattenuating nodule in liver (arrow), **(C)** Early PET image and **(D)** Corresponding PET/CT fusion image show that the nodule does not show obvious hypermetabolism. **(E)** Fat-suppressed T2WI shows two hyperintense nodules in liver (arrows). **(F)** DW image (b = 800 sec/mm2) shows two nodules with restricted diffusion (arrows). **(G)** Delayed PET image shows two hypermetabolic nodules in liver (arrows). **(H)** Corresponding PET and DW fusion image shows the two nodules with both hyperintense and hypermetabolism (arrows).

Of the 40 patients, 14 patients had liver metastases (see **Table 4**; **Figures 3**, **4**). For the lesion-based analysis, the number of liver metastases detected by CE-CT/MR imaging, PET/CT and PET/MR imaging were 33, 18, and 61, respectively. For the patient-based analysis, compared with CE-CT/MR imaging, PET/MR imaging resulted in additional findings of more metastases in 9/14 patients. Specifically, 3/14 patients with liver metastases were upstaged. Compared with PET/CT, PET/MR imaging resulted in additional findings of more metastases in 12/14 patients, of which 6 patients were upstaged.

**Table 4:** Diagnostic performance of PET/CT plus delayed PET/MR imaging and CE-CT/MR imaging in the detection of liver metastases.

Patients	CE-CT/MR		PET/CT		PET/MR		CE-CT/MR vs PET/MR		PET/CT vs PET/MR	
	M stage	Number	M stage	Number	M stage	Number	Additional finding in PET/MR	Staging change	Additional finding in PET/MR	Staging change
1	1	3	1	3	1	3	None	None	None	None
2	1	6	1	3	1	12	More metastases	None	More metastases	None
3	1	2	0	0	1	2	None	None	More metastases	Up
4	1	5	1	1	1	5	None	None	More metastases	None
5	1	1	0	0	1	2	More metastases	None	More metastases	Up
6	1	1	0	0	1	3	More metastases	None	More metastases	Up
7	1	4	1	2	1	5	More metastases	None	More metastases	None
8	0	0	0	0	1	6	More metastases	Up	More metastases	Up
9	0	0	0	0	1	2	More metastases	Up	More metastases	Up
10	1	4	1	2	1	6	More metastases	None	More metastases	None
11	1	1	0	0	1	1	None	None	More metastases	Up
12	1	2	1	1	1	3	More metastases	None	More metastases	None
13	0	0	1	2	1	7	More metastases	Up	More metastases	None
14	1	4	1	4	1	4	None	None	None	None
Sum	11	33	8	18	13	61	9	3	12	6

## Discussion

In this prospective study, we demonstrated that nonenhanced whole-body FDG PET/CT plus delayed abdomen PET/MR imaging showed similar diagnostic performance without a statistically significant difference in the assessment of the tumor resectability and M stage of pancreatic tumors compared with the widely used CE-CT/MR imaging. Excitedly, based on the combination of size and normalized SUVmax of lymph nodes, PET/CT plus delayed PET/MR imaging showed higher sensitivity than CE-CT/MR imaging, with a statistically significant difference. What is more, the number of total liver metastases detected by delayed PET/MR imaging was nearly twice of that of CE-CT/MR imaging. Although this study is only exploratory, with a small number of patients, to our knowledge, the findings are the first to suggest that the combination of nonenhanced whole-body FDG PET/CT and delayed abdomen PET/MR imaging may be a more reasonable examining approach for the preoperative evaluation of pancreatic cancer, hopefully substituting for the widely used CE-CT and leading to improvement in creating a more efficient work-up flow. Indeed, Raman et al. (26) have reported that the accuracy of multidetector CT in excluding distant metastatic disease in patients with pancreatic cancer significantly depreciates over time because the tumor can metastasize during the interval between multidetector CT and surgery. Therefore, FDG PET/CT plus delayed PET/MR imaging may play a valuable role in simplifying the work-up flow and shortening the work-up period of pancreatic tumors, avoiding conversion from resectable status to unresectable status due to the rapidly progressive characteristic of pancreatic tumors.

Patients with pancreatic cancer could benefit from upfront pancreatic resection when achieving a curative resection with negative margins; thus, precise preoperative assessment of tumor resectability is vital (27). Pancreatic cancer resectability is determined primarily by the degree of tumor–vascular contact and distant metastasis (22, 23). In our study, no significant differences of evaluating tumor resectability were observed between PET/CT plus delayed PET/MR imaging and CE-CT/MR imaging. For the evaluation of the presence and/or extent of vascular involvement, CE-CT, with its superior spatial resolution and ability to perform multiplanar and 3D reconstructions to depict vascular involvement, has been regarded as the best method to determine surgical resectability (3, 28). However, in our study, based on the blood flowing void effect at 4 mm-slice T2WI, nonenhanced PET/MR imaging and CE-CT/MR imaging had equivalent diagnostic performance in terms of vascular invasion. Considering that most of our patients with resectable or borderline resectable pancreatic tumors, our study performance may actually have been overestimated. Admittedly, for the evaluation of distant metastasis, PET/MR imaging combines the excellent soft-tissue contrast of MR imaging with the high sensitivity of PET, enabling the depiction of subtle metastatic lesions, which can directly upstage patients from potentially resectable status to metastatic unresectable status. Thus, we considered that nonenhanced PET/CT plus delayed PET/MR imaging has potential as a substitute for CE-CT/MR imaging in assessment of resectability, certainly, which still remains a large sample of research.

Accurate assessment of lymph node metastases in patients with pancreatic cancer plays an important role in the prediction of a patient’s prognosis (29). In our study, which used imaging criteria of size (shortest diameter >8 mm) and PET positivity (normalized SUVmax >1) for N staging, PET/CT plus delayed PET/MR imaging showed higher sensitivities than common CE-CT/MR imaging with a statistically significant difference. The result suggests that PET/CT plus delayed PET/MR imaging, which provides both anatomic and metabolic information, can be useful in the detection and characterization of metastatic lymph nodes. However, our preliminary study failed to demonstrate a significant difference between PET/CT plus delayed PET/MR imaging and CE-CT/MR imaging in the specificity and accuracy. This can be attributed to the limitation of size-based assessment that reactive lymph nodes can be enlarged and small lymph nodes can have micrometastases (30). In addition, PET also had limited performance in the detection of lymph node metastases because PET positivity can also be found in the inflammatory and anthracosilicostic nodes (31). Notably, we are the first to select the either parameter of normalized SUVmax from PET/CT or delayed PET/MR imaging to evaluate lymph node metastases, with a good result in the higher sensitivities. Thus, PET/CT plus delayed PET/MR imaging has potential as a valuable tool for N staging and future studies with a larger population are warranted.

As for M staging, most commonly, metastatic disease from pancreatic cancer is observed in the liver (32). Thus, liver metastases in patients with pancreatic cancer should raise suspicion of M1 disease and then, the change from M0 to M1 can directly result in a change from resectability to unresectability. In our study, although diagnostic performance did not significantly differ between PET/CT plus delayed PET/MR imaging and CE-CT/MR imaging in our study, the number of liver metastases detected by delayed PET/MR imaging was nearly twice that of CE-CT/MR imaging and three times that of PET/CT. Although PET/CT was considered as an ideal imaging modality to detect distant metastases that may be missed using other modalities, the study by Fröhlich et al. (33) indicated that PET/CT has high sensitivity (97%) in detecting metastases larger than 1 cm in diameter, sensitivity falls to 43% for smaller lesions, which may be the reason for the less liver metastases detected by PET/CT than PET/MR imaging and CE-CT/MR imaging. However, in the light of the fact that noncontrast MR imaging has far superior soft tissue discrimination compared with noncontrast CT and has also been found to be superior to CT in the detection of liver metastases with a sensitivity of 90%–93% (34), we believed that PET/MR imaging can make up for the disadvantage of PET/CT. Notably, the PET imaging performance of delayed PET/MR is also better than that of PET/CT, which is different from the previous results that the PET imaging performance of PET/MR imaging would be similar to that of PET/CT (7, 35, 36). This may be the foremost reason for that delayed PET/MR imaging had the largest number of liver metastases of the three imaging systems. Our result demonstrated delayed PET/MR imaging has a potential as the most valuable imaging modality for the detection of liver metastases on the basis of the good performance of both delayed PET and MR imaging, especially for the delayed PET imaging performance, which can be conducive to accurate M staging.

This study had several limitations. First, the number of patients in this prospective study is relatively small, so these first results have to be considered preliminary and need further confirmation. Second, we could not match the imaging-based diagnosis of vascular involvement and/or lymph node status with the corresponding pathological results, so we assessed the resectability and staging of pancreatic tumors on a patient-by-patient basis rather than on a lesion-by-lesion basis. Third, given that the imaging features of pancreatic tumor reported by numerous studies, we did not evaluate the size, conspicuity and PET-related parameters of pancreatic tumor, and did not compare PET-related parameters at PET/CT and delayed PET/MR imaging.

In conclusion, nonenhanced whole-body FDG PET/CT plus delayed abdomen PET/MR imaging showed comparable diagnostic performance with CE-CT/MR imaging in the evaluation of the resectability and staging of pancreatic cancers; furthermore, it provided additional value of detecting liver metastases, which still has potential as the more efficient and reasonable work-up approach.

## Data Availability Statement

The original contributions presented in the study are included in the article/supplementary material. Further inquiries can be directed to the corresponding author.

## Ethics Statement

This study was approved by the 76 Medical Ethics Committee of Peking University Cancer Hospital (ethical approval No. 2018KT110-77 GZ01). All patients provided signed informed consent before the examinations.

## Author Contributions

ZZ and NZ substantially contributed to designing the study and drafting the manuscript. XG and HZ contributed to the acquisition, analysis, or interpretation of the data. NL and ZY revised it critically for important intellectual content. ZY finally approved the version to be published and agreed to be accountable for all aspects of the work in ensuring that questions related to the accuracy or integrity of any part of the work are appropriately investigated and resolved. All authors contributed to the article and approved the submitted version.

## Funding

The current research was financially supported by the Science Foundation of Peking University Cancer Hospital (No. 2021-4) and Beijing Municipal Administration of Hospitals, Yangfan Project (ZYLX201816).

## Conflict of Interest

The authors declare that the research was conducted in the absence of any commercial or financial relationships that could be construed as a potential conflict of interest.

## Publisher’s Note

All claims expressed in this article are solely those of the authors and do not necessarily represent those of their affiliated organizations, or those of the publisher, the editors and the reviewers. Any product that may be evaluated in this article, or claim that may be made by its manufacturer, is not guaranteed or endorsed by the publisher.
